# Disordered metabolism in mice lacking irisin

**DOI:** 10.1038/s41598-020-74588-7

**Published:** 2020-10-15

**Authors:** Yunyao Luo, Xiaoyong Qiao, Yaxian Ma, Hongxia Deng, Charles C. Xu, Liangzhi Xu

**Affiliations:** 1 Reproductive Endocrinology and Regulation Laboratory West China Second University Hospital, Sichuan University, #20 Section 3, Ren Min Nan Road, Chengdu, 610041 Sichuan People’s Republic of China; 2 The Joint Laboratory for Reproductive Medicine of Sichuan University, The Chinese University of Hong Kong, Hong Kong, People’s Republic of China; 3 Key Laboratory of Birth Defects and Related Diseases of Women and Children (Sichuan University), Ministry of Education, Chengdu, People’s Republic of China; 4 Department of Obstetrics and Gynecology, West China Second University Hospital, Sichuan University, Chengdu, People’s Republic of China; 5 College of Engineering, The Ohio State University, Columbus, OH USA

**Keywords:** Biomarkers, Endocrinology

## Abstract

Irisin is a product of fibronectin type III domain-containing protein (*Fndc5*) and is involved in the regulation of adipokine secretion and the differentiation of osteoblasts and osteoclasts. In this study, we aimed to determine whether irisin lacking affects glucose/lipid and bone metabolism. We knocked out the *Fndc5* gene to generate irisin-lacking mice. Remarkable, irisin lacking was related to poor ‘browning response’, with a bigger size of the intraperitoneal white adipose cell and decreased a number of brown adipose cells in brown adipose of interscapular tissue. The irisin lacking mice had hyperlipidemia and insulin resistance, reduced HDL-cholesterol level, increased LDL-cholesterol level, and decreased insulin sensitivity. The lacking of irisin was associated with reduced bone strength and bone mass in mice. The increased number of osteoclasts and higher expression of RANKL indicated increased bone resorption in irisin lacking mice. The level of IL-6 and TNF-α also increased in irisin lacking mice. The results showed that irisin lacking was related to decreased ‘browning response’, glucose/lipid metabolic derangement, and reduced bone mass with increased bone resorption. Further studies are needed to confirm these initial observations and explore the mechanisms underlying the effects of irisin on glucose/lipid and bone metabolism.

## Introduction

Irisin is a recently described cytokine secreted by skeletal muscles and was discovered in 2012^1^. It is derived from the 196 amino acid transmembrane protein fibronectin type III domain containing 5 (*Fndc5*), which is cleaved by specific proteases^2,3^. Fndc5 is completely conserved among vertebrates^1^. Irisin is an identified myokine that may play an important metabolic role in regulating adipose tissue metabolism by converting white to brown adipose tissue^4^. Recently, some studies have shown that serum irisin levels are correlated with metabolic diseases, including type 2 diabetes (T2D), obesity, and metabolic syndrome^5,6^. Exogenously administered irisin induces energy expenditure and weight loss and improves insulin resistance in high-fat-fed mice^1^. Xiong et al*.* suggested that the deletion of *Fndc5*, worsened obesity, and exacerbated insulin resistance in male mice^7^.

More data also suggested the potential effect of irisin in bone metabolism. Bone metabolism is a dynamic bone remodeling process performed by osteoblasts and osteoclasts and includes bone formation and resorption. Imbalanced bone remodeling leads to osteoporosis, a highly prevalent and severe public health concern^8,9^. Colaianni et al. indicated that irisin injection prevented bone loss and induced recovery of bone mass in 2-month-old male mice^10^. These effects were consistent with in vitro studies in our lab, indicating that irisin enhances osteoblast differentiation but inhibits osteoclast precursor cell differentiation^11,12^. Furthermore, our previous studies also revealed that intraperitoneal injection of irisin reduced bone loss in ovariectomized (OVX) mice, principally due to a marked reduction in bone resorption^13^.

Thus, we knocked out the *Fndc5* gene to generate irisin lacking mice, to identify the multiple effects of irisin in metabolism.

## Materials and methods

This study was approved by the Ethics Committee of West China Second University Hospital, Sichuan University, China. We confirmed that all experiments were performed in accordance with relevant guidelines and regulations.

### Reagents

The enzyme-linked immunosorbent assay (ELISA) kit for osteocalcin (OCN) (E06917m), Alkaline Phosphatase (ALP) (E0200m), and Tartrate-resistant acid phosphatase (TRAP) (E08492m) were purchased from Elabscience (Wuhan, China). The TRAP staining solution was purchased from Sigma-Aldrich (N0378, USA). Antibody against osteoprotegerin (OPG) (ab9986) and receptor activator of nuclear factor-kB ligand (RANKL) (ab216484) were purchased from Abcam (Cambridge, MA, USA). The low-density lipoprotein cholesterol assay kit (CSB-E13476m), high-density lipoprotein cholesterol assay kit (CSB-E12874m) were purchased from CUSABIO (Wuhan, China). The total cholesterol assay kit (E-BC-K109-S), and triglyceride assay kit (E-BC-K261-M) were purchased from Elabscience (Wuhan, China).

### Fndc5 knockout mice

**Figure 1:**
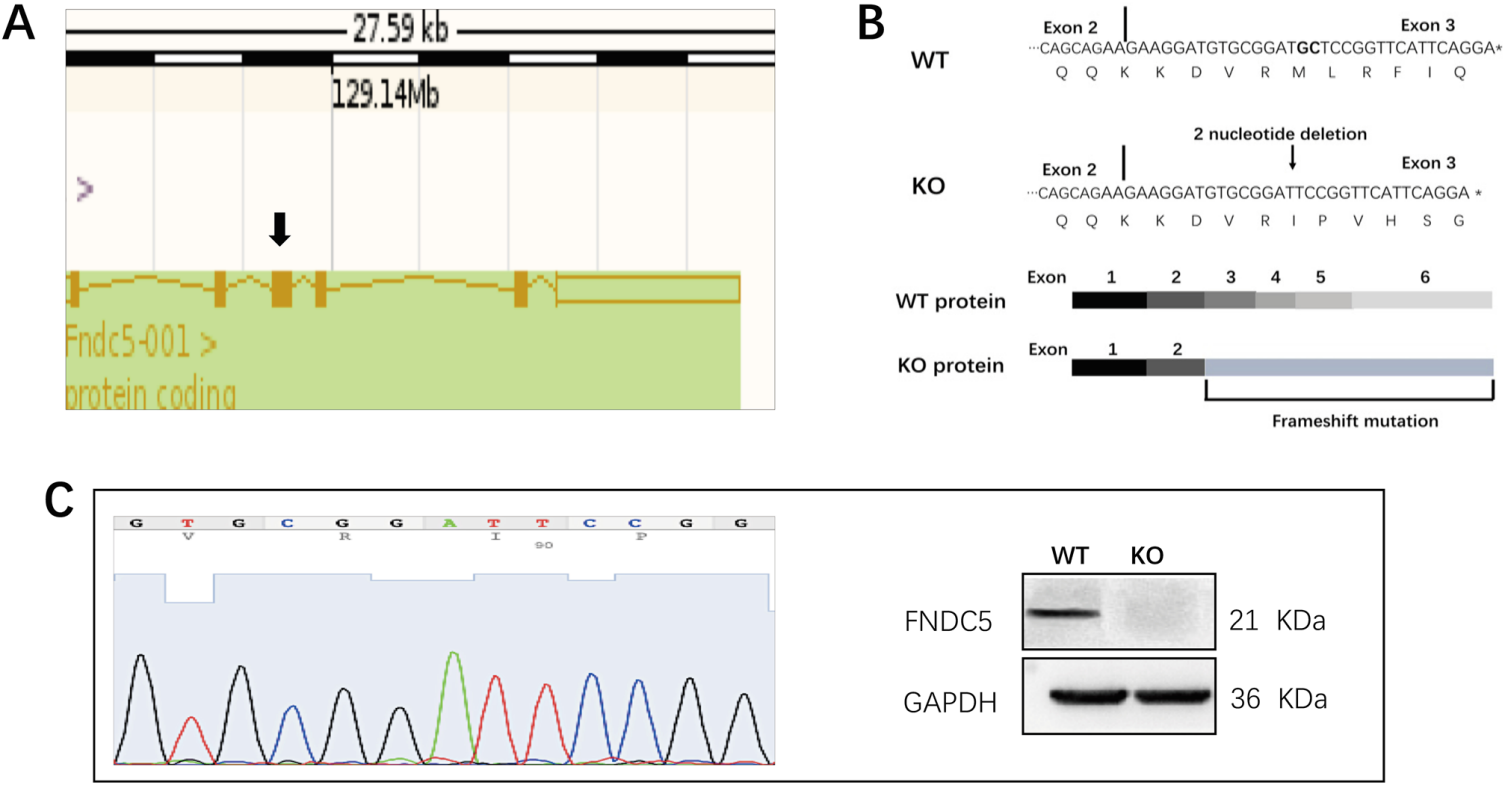
Generation of Fndc5 KO mice. (**A**) Schematic representation of the gene targeting procedure. The black arrowhead indicates the target exon. (**B**) Sequences of the *Fndc5* mRNA transcripts in WT and KO mice and the proteins predicted to be encoded by the respective mRNAs (Chormas v2.1.3, https://www.lhdown.com/soft-/28807.html). (**C**) Confirmation of *Fndc5* knockout. Genotyping (left panel) and Western blot (right panel) analyses were consistent with successful *Fndc5* knockout (Quantity One, v4.6.6, https://www.opdown.com/soft/9293-3.html).

Irisin is produced by proteolytic processing of a transmembrane receptor. *Fndc5* is a 209-residue protein with an N-terminal 29-residue signal sequence followed by the irisin or putative fibronectin III domain, a linking peptide, a transmembrane domain, and a 39-residue cytoplasmic segment. Cleavage in the linking peptide region releases soluble irisin into the extracellular milieu. Therefore, we knocked out the *Fndc5* gene to generate irisin lacking mice. Female *Fndc5*-heterozygous (+/−) mice were generated by View solid-biotech, Inc. (Beijing, China). Transcription activator-like effector nuclease (TALEN) technology was used to shear the DNA encoding the exon of the target gene. *Fndc5* has six exons, and the coding gene of irisin is located in exon 3. Clipping the 18th and 19th nucleotides in exon three led to a frameshift mutation, which induced irisin deficiency in mice (Fig. 1A,B). *Fndc5*-heterozygous (+/−) female mice were bred with C57BL/6 WT male mice (Dossy, Chengdu, China) to produce heterozygous (+/−) male mice. Subsequently, female *Fndc5*-deficient (−/−) mice (Fig. 1C) were created by mating heterozygous (+/−) female mice with heterozygous (+/−) male mice. PCR-based genotyping analysis with tail genomic DNA was performed for *Fndc5* using the following primers: 5′-CATGTTTCCTTAGCTCTACTGTG-3′ (forward) and 5′-GGAGAAAGCATGCATGGCAGTCT-3′ (reverse). It took nearly 2 years to generate 51 homozygous mice. Mice were housed with 4 to 5 animals per cage in a temperature-controlled environment on a 12-h light/dark cycle. All animals were given access to food (Dossy company, Chengdu, China) and water. After 24 weeks of old, the animals were sacrificed. The liver, spleen, kidney, intraperitoneal white adipose tissue (iWAT), and interscapular brown adipose tissue (iBAT) were weighted, and blood samples were collected the obit for serum isolation. Photomicrographs of hematoxylin and eosin-stained sections of iWAT and iBAT were analyzed with ImageJ software (NIH, MD) for measuring adipocytes size and area. The total adipocytes area was measured in the number of pixels and then converted to the metric system. The femur and tibia were dissected and divested of soft tissue for histology, microarchitecture analysis, and a three-point bending test.

### GTTs and ITTs

Glucose and insulin tolerance tests were performed on mice after 12 h of fasting. The 24-weeks-old mice received an intraperitoneal injection with dextrose solution (1 g/kg, West China Second University Hospital, SCU, China) for GTTs or with insulin (0.35 U/kg, West China Second University Hospital, SCU, China) for ITTs. Blood was drawn at 0, 30, 60, 90, and 120 min after injection for blood glucose determination using an Easy Step blood glucose monitor [Sensitivity 1.1–33.3 mmol/L] (Yu Yue Medical Company, China).

### Three-point bending test

The tibia was dissected and separated from adjacent tissues. All specimens were tested under a load applied at a constant rate of 20 mm/min to analyze the biomechanical strength at the mid-portion of the tibia using a three-point bending test conducted with a bone strength testing device (Bose 3220 Series II, Massachusetts, USA) as described previously^14^.

### Analysis of bone microarchitecture

We assessed trabecular microarchitecture in the distal femur by micro-computed tomography (μCT, Quantum GX, PerkinElmer, USA). The transverse μCT slices were evaluated in an area 1–3 mm to the distal growth plate. The X-ray source was set at a voltage of 90 kVp and a current of 88 μA, with a 0.5-mm aluminum filter. The scanning angular rotation was 180^。^, and the volume size was set at 10 μm. The trabecular bone region was identified by semi-manually contouring the trabecular bone in the ROI with the assistance of an auto-thresholding software algorithm (Analyze 12.0, PerkinElmer). Morphometric variables were computed from the binarized images using direct, 3D techniques we assessed the bone mineral density (BMD; g/cm^2^), trabecular bone volume (bone volume to tissue volume ratio, BV/TV; %), trabecular number (Tb. N; mm^−1^), and connection density (Conn.D; 1/mm^3^).

### TRAP staining

The distal femur was fixed in 4% PFA fixation solution. After decalcifying in ethylene diamine tetra-acetic acid (EDTA) for seven days, the femur was longitudinally sectioned (5 μm thickness), and the sections were stained for TRAP. The cells expressing TRAP enzyme were stained pink^15^. The slides were scanned with a Nikon ECLIPSE Ti Slide Scanning System (Nikon, Japan), and image analysis was performed using ImageJ software (NIH, MD).

### Immunohistochemistry

Femur sections were pretreated with 0.1% Tween in PBS for 30 min. Tissue sections were blocked in 5.0% BSA for 2 h before incubation with 5 μg/ml rabbit polyclonal antibody against OPG or RANKL overnight at 4 ℃. Then, the tissue sections were washed in TBST and incubated with HRP-conjugated secondary antibodies (Zen-Bioscience Company, China). The color was developed using diaminobenzidine (DAB) peroxidase substrate (Invitrogen, UK) for 2 min before counterstaining with hematoxylin (Solarbio, Beijing, China). Images were analyzed with the ImageJ software (NIH, MD) for quantifying the intensity of the reaction (Mean density = IOD Sum/Area Sum).

### Serum levels of bone markers

The OCN, and TRAP levels in serum samples were measured using the ELISA kit (Elabscience, Wuhan, China). The ELISA assays were performed according to the manufacturer’s manual, respectively.

### Cytokine measurement by electrochemiluminescence assay

The MSD V-PLEX Proinflammatory Panel 1 Mouse Kit (Rockville, Maryland, USA) was used to measure plasma interferon-gamma (IFN-γ), interleukin 10 (IL-10), interleukin 12p70 (IL-12), interleukin 1-beta (IL-1β), interleukin 2 (IL-2), interleukin 4 (IL-4), interleukin 5 (IL-5), interleukin 6 (IL-6), growth-regulating oncogenes (GRO), and tumor necrosis factor-alpha (TNF-α) concentrations in individual samples. We performed this assay using 50 μl from each sample. Standard volumes of 25 μl of plasma were utilized for each panel and diluted twofold using the assay diluent according to the package insert. All samples were run in duplicate and processed according to the manufacturer’s instructions.

### Statistical analysis

All data are presented as the mean ± SEM. Differences in the mean values between two groups were assessed by two-tailed Student t-test. One-way and two-way ANOVA were used for data analysis of more than two groups, followed by Bonferroni’s post hoc analysis. *P* < 0.05 was considered statistically significant.

## Results

### Reduced browning response and hyperlipidemia in irisin lacing mice

**Figure 2:**
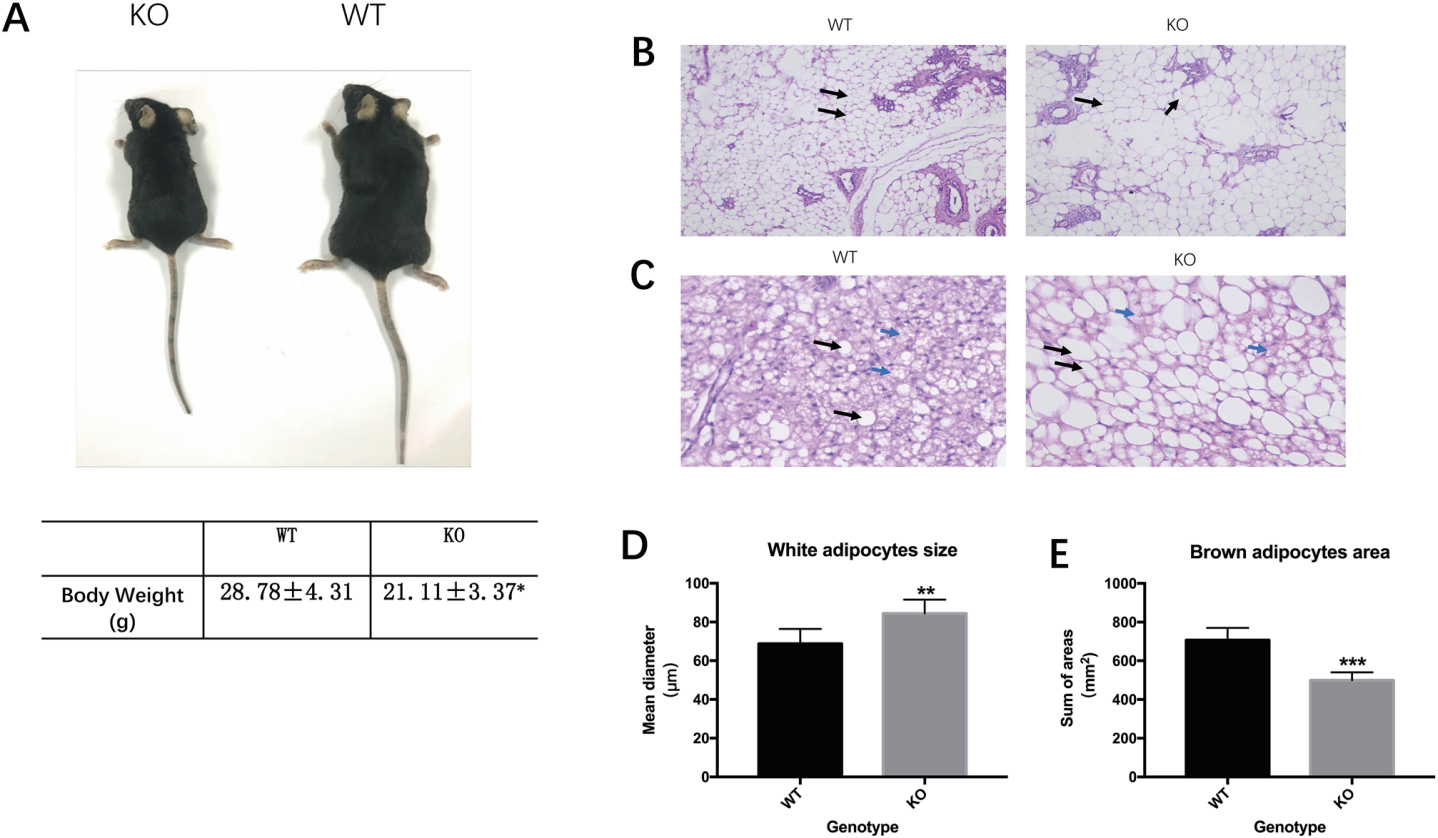
Poor ‘browning response’ in irisin lacking mice. (**A**) Appearance of WT and KO mice; (**B**, **C**) representative sections of H&E staining showing adipocytes in intraperitoneal white adipose tissue (iWAT) and interscapular brown adipose tissue (iBAT) (100×) (NIS-Elements Viewer, v4.2.0; https://www.downza.cn/soft/275121.-html); (**D**) the mean diameter of white adipose cells in iWAT. (**E**) The sum of the brown adipose area in iBAT (Graphpad Prism, v7.0, https://www.xue51.com/soft/393-2.html). Data are presented as the mean ± SEM, n = 10 per group, **P < 0.01; ***P < 0.001 compared to the WT group.

In this study, we knocked out *Fndc5* to generated irisin lacking mice (KO), and performed wild type mice (WT) as a control group. Although KO mice had significantly reduced body weight, they had an increased intraperitoneal white adipose tissue (iWAT) ratio, and a decreased interscapular brown adipose tissue (iBAT) ratio compared to WT mice (Fig. 2A; Table 1).

**Table 1:** The general conditions of mice in the different groups. intraperitoneal white adipose tissue (iWAT), interscapular brown adipose tissue (iBAT). The ratio = tissue weight/body weight. Data represent the mean ± SEM (n = 20). *P < 0.05 compared to WT.

	WT	KO
Body Weight (g)	28.78 ± 4.31	21.11 ± 3.37*
iWAT ratio (%)	0.0187 ± 0.01	0.0269 ± 0.01*
iBAT ratio (%)	0.011 ± 0.0002	0.007 ± 0.0003*
Liver ratio (%)	0.054 ± 0.01	0.058 ± 0.01
Spleen ratio (%)	0.070 ± 0.0002	0.062 ± 0.0002
Kidney ratio (%)	0.034 ± 0.01	0.036 ± 0.01

**Figure 3:**
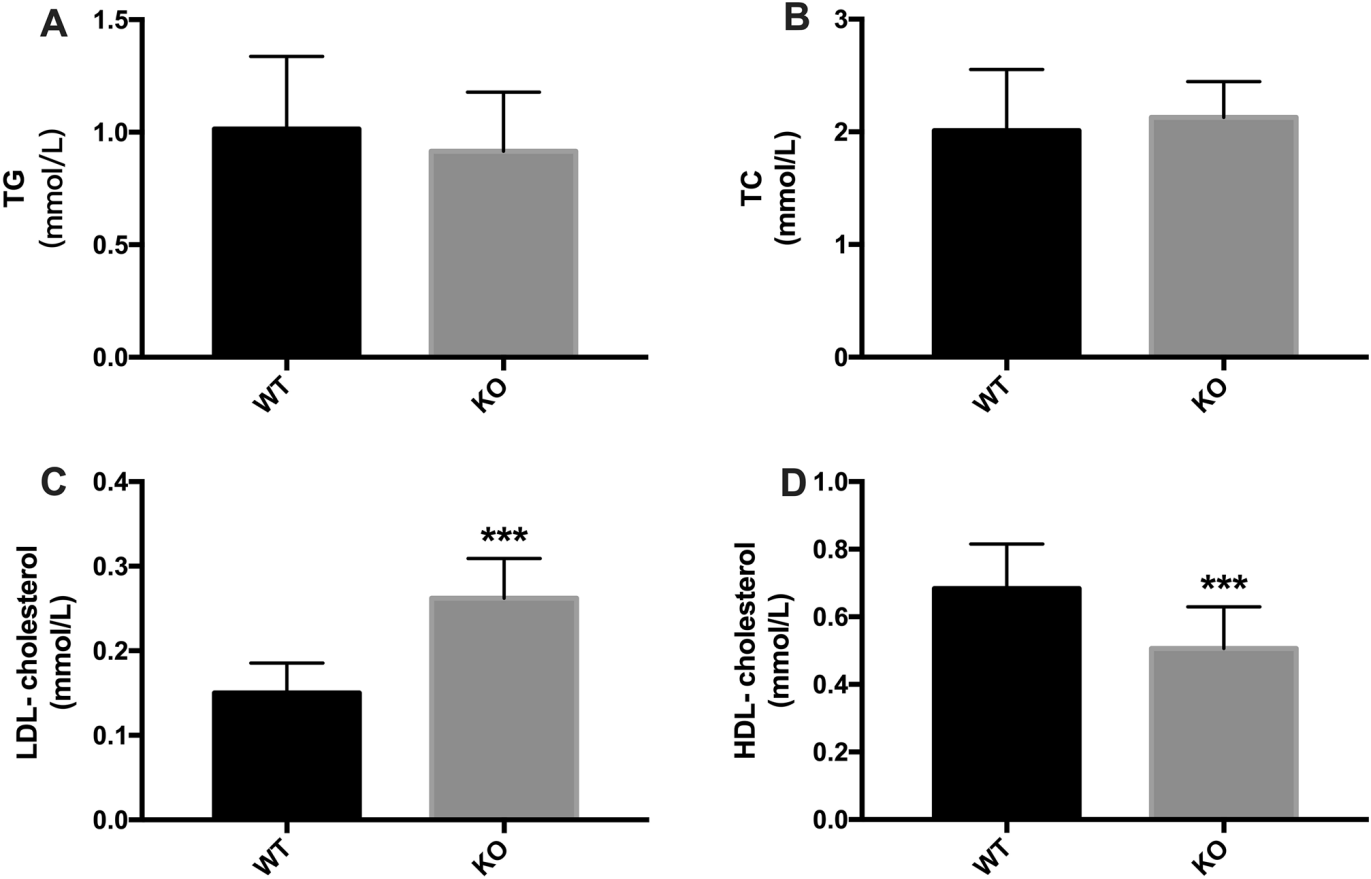
TG, TC, LDL-cholesterol, and HDL-cholesterol concentrations in serum. Data are presented as the mean ± SEM, n = 15 per group, with three replicates. (Graphpad Prism, v7.0, https://www.xue51.com/soft/3932.html). ***P < 0.001 compared to the WT group.

Besides, we stained adipose sections with H&E, and noted that KO mice had a bigger size of white adipocyte size (Fig. 2B,D). There was a weak ‘browning response’ in KO mice, displayed by fewer brown adipocytes in adipose tissue (Fig. 2C,E). To identify the effects of irisin on lipid metabolism, we measured the blood lipids of the mice. The results showed that KO mice had higher LDL-cholesterol levels (0.26 ± 0.02 mmol/L vs 0.15 ± 0.01 mmol/L, *P* < 0.001) and lower HDL-cholesterol levels (0.51 ± 0.04 mmol/L vs 0.68 ± 0.04 mmol/L, *P* < 0.001) compared to WT mice (Fig. 3C,D). The TG and TC concentrations were similar between the two groups (Fig. 3A,B). The liver, spleen, and kidney weights were comparable between the two groups (Table 1).

### Reduced glucose tolerance and insulin sensitivity in irisin lacking mice

**Figure 4:**
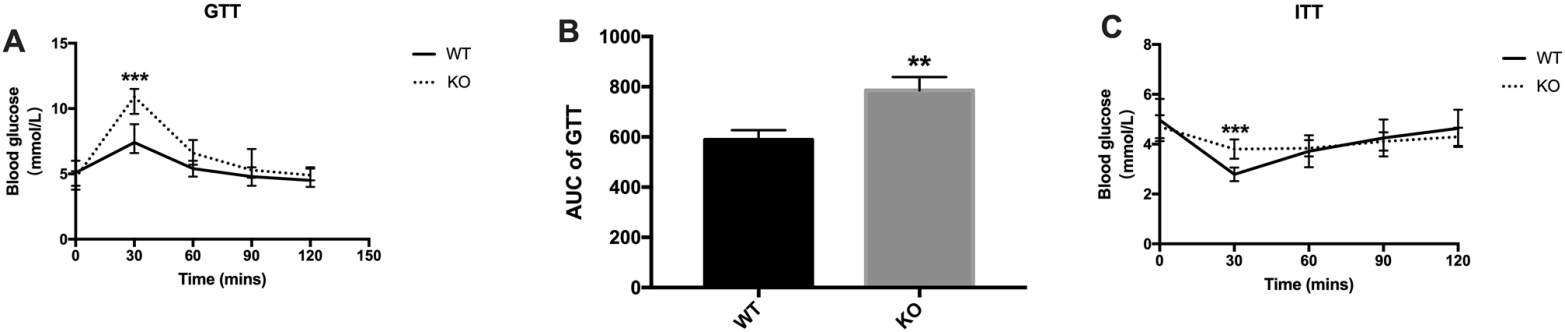
Reduced glucose tolerance and insulin sensitivity in irisin lacking mice. (**A**) Glucose tolerance test (GTT); (**B**) area under curve (AUC) of the GTT; (**C**) insulin tolerance test (ITT). Data are presented as the mean ± SEM, n = 15 per group, (Graphpad Prism, v7.0, https://www.xue51.com/soft/3932.html). **P < 0.01, ***P < 0.001 compared to WT group.

We performed a glucose tolerance test (GTT) and an insulin tolerance test (ITT) to evaluate glucose tolerance and insulin sensitivity. Our results showed that KO mice had poor glucose tolerance and significantly elevated glucose levels at 30 min following glucose challenge compared with those of WT mice (10.78 ± 0.25 mmol/L vs. 7.08 ± 0.22 mmol/L, *P* < 0.001) (Fig. 4A,B). We quantified the efficiency of insulin by its ability to reduce blood glucose levels in the ITT. The results showed that KO mice had decreased insulin sensitivity (3.80 ± 0.11 mmol/L vs. 2.79 ± 0.10 mmol/L, *P* < 0.001), showing insulin resistance (Fig. 4C).

### Reduced bone strength and bone mass in irisin lacking mice

To investigate the role of irisin in bone metabolism, we measured tibia bone strength using a three-point bending test. The KO mice displayed a decline in bending load (max power to induce deformity in the bone) by ~ 39% (*P* < 0.01) and stiffness (amount of rigidity in the bone) by 27% (*P* < 0.01) when compared to WT mice (Table 2).

**Table 2:** Biomechanical strength of the tibia and three-point bending parameters in different groups. Data represent the mean ± SEM (n = 15). **P < 0.01 compared to WT.

Biomechanical parameters	WT	KO
Maximum bending load (N)	25.71 ± 18.78	15.60 ± 6.31**
Bending displacement (mm)	2.25 ± 0.87	2.03 ± 0.76
Stiffness (N/mm)	11.30 ± 5.06	8.29 ± 3.22**

**Figure 5:**
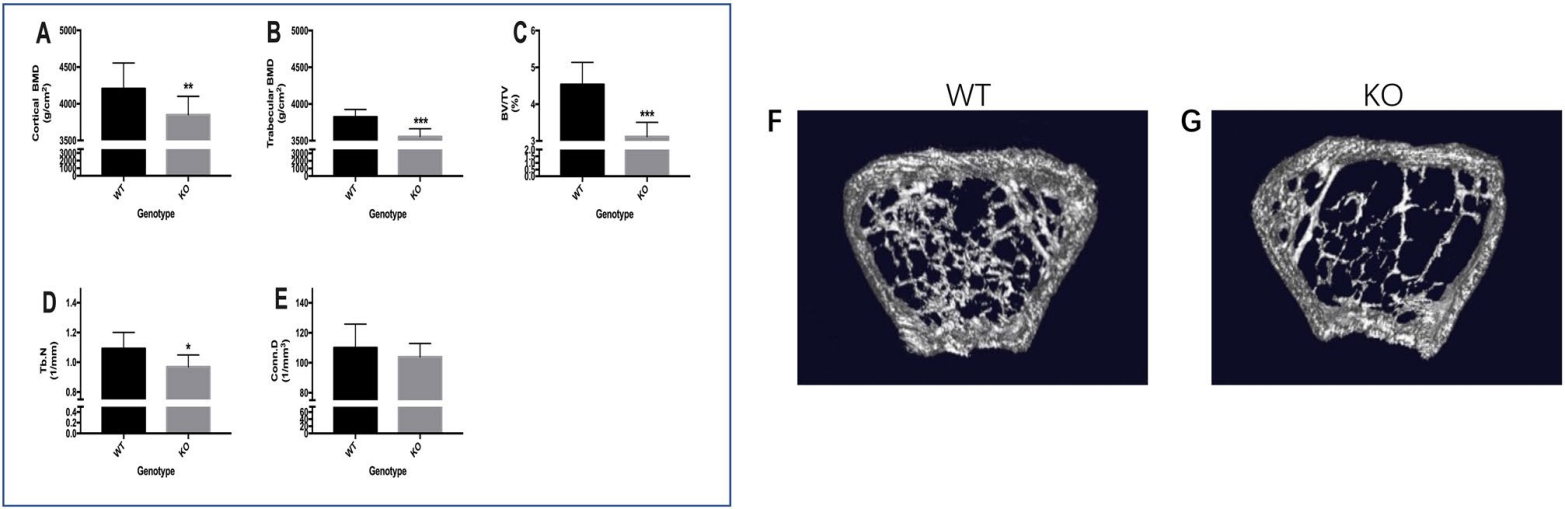
Decreased bone mass in irisin lacking mice. Bone morphological parameters measured by μCT. (**A**–**E**) Cortical bone mineral density (cBMD), trabecular bone mineral density (tBMD), trabecular volume/tissue volume (BV/TV), trabecular number (Tb. N) and connection density (Conn. D) (Graphpad Prism, v7.0, https://www.xue51.com/soft/3932.html); (**F**,**G**) 3D images of the femur microstructure in the two groups (Analyze, v12.0, https://analyzedirect.com/training-guide/). Data are presented as the mean ± SEM from n = 22 WT mice and n = 21 KO mice. *P < 0.05, **P < 0.01, ***P < 0.001 compared to WT group.

The femur of each animal was subjected to μCT scanning for the evaluation of bone microstructure. The results showed that KO mice had decreased bone mass at 24 weeks of age, with lower cortical bone mineral density (cBMD) (3850 ± 70.17 g/cm^2^ vs 4295.97 ± 97.15 g/cm^2^ , *P* < 0.01), lower trabecular bone mineral density (tBMD) (3555 ± 40.87 g/cm^2^ vs 3823 ± 36.21 g/cm^2^, *P* < 0.001), lower bone volume/total volume (BV/TV) (3.12 ± 0.15% vs 4.44 ± 0.30%, *P* < 0.001), and lower trabecular number (Tb. N) (0.87 ± 0.14 mm^−1^ vs 1.10 ± 0.16 mm^−1^, *P* < 0.05) compared to WT mice (Fig. 5A–D). The connection density (Conn. D) was comparable in the two groups (Fig. 5E). Furthermore, the 3D images showed a consistent trend in bone microarchitecture (Fig. 5F,G).

**Figure 6:**
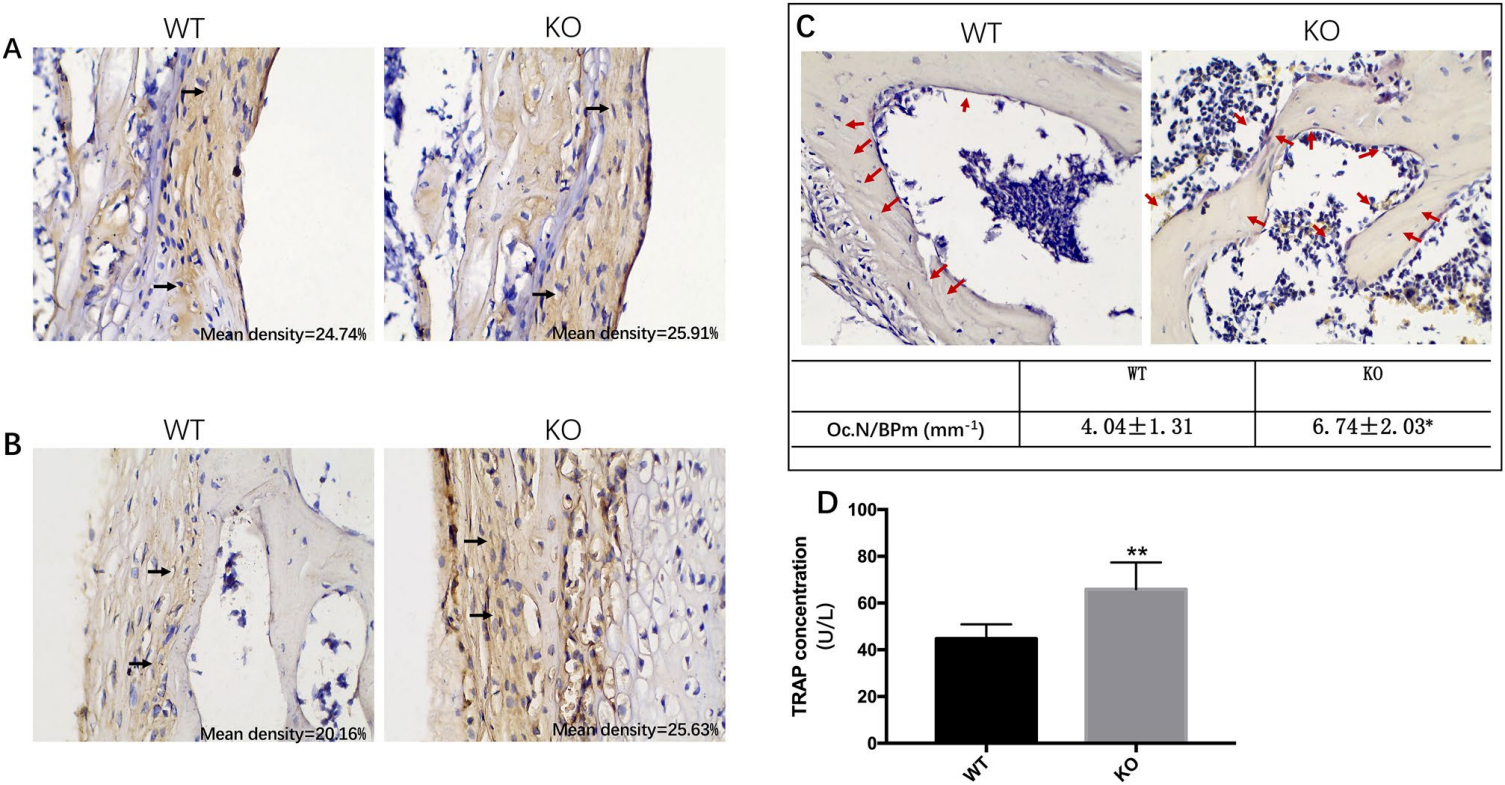
Increased bone resorption in irisin lacking mice. (**A**, **B**) Sections of the distal shaft of the femur were stained with osteoprotegerin (OPG) (1:60) and receptor activator of nuclear factor-kB ligand (RANKL) (1:80) antibodies. Positive (black arrows) osteoblasts are shown, 200×. (**C**) Representative images of the distal metaphyseal region of the femur, together with cell counts (osteoclasts) per bone perimeter (Bpm). Red arrows, tartrate-resistant acid phosphatase (TRAP)-positive osteoclasts, 100×, (NIS-Elements Viewer, v4.2.0; https://www.downza.cn/soft/2751-21.html); (**D**) serum levels of osteocalcin TRAP (with three replicates). (Graphpad Prism, v7.0, https://www.xue51.com/soft/3932.html). Data are presented as the mean ± SEM (n = 15 per group). *P < 0.05, **P < 0.01 compared to the WT group.

**Figure 7:**
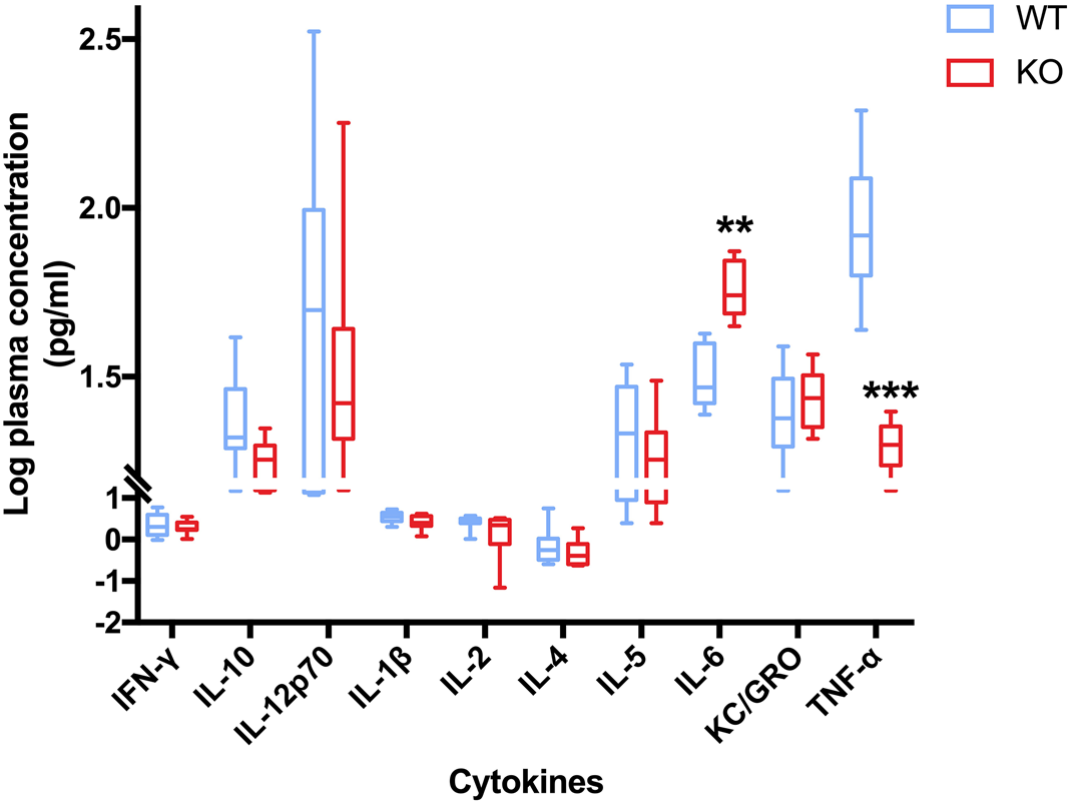
The distribution of the log of transformed cytokine concentrations. The figure represents the distributions of interferon-gamma (IFN-γ), interleukin 10 (IL-10), interleukin 12p70 (IL-12), interleukin 1-beta (IL-1β), interleukin 2 (IL-2), interleukin 4 (IL-4), interleukin 5 (IL-5), interleukin 6 (IL-6), growth-regulating oncogenes (GRO), and tumor necrosis factor-alpha (TNF-α). (n = 15 per group, with three replicates). (Graphpad Prism, v7.0, https://www.xue51.com/soft/3932.html). **P < 0.01, *P* < 0.001 compared to the WT group.

### Increased bone resorption in irisin lacking mice

In general, decreased bone mass is associated with decreased osteoblastic bone formation and, or increased osteoclastic bone resorption. OPG and RANKL are known to mediate osteoclast genesis. We investigated the dynamic bone resorption parameters in the distal femur. The results showed that KO mice had higher RANKL expression, with intense labeling (Fig. 6B), and the OPG signal were similar in KO and WT group (Fig. 6A). Also, we further investigated bone resorption parameters based on TRAP staining. Our results showed that KO mice had increased osteoclast numbers (Fig. 6C). In addition, we measured the bone formation and resorption parameters in serum, and the results indicated that KO mice had a higher TRAP level than WT mice (65.86 ± 4.70 U/L vs. 44.77 ± 2.48 U/L, *P* < 0.01) (Fig. 6D). The serum levels of OCN and ALP were comparable in KO and WT mice (Supplementary Figure 1).

### Increased proinflammatory cytokines level in irisin lacking mice

The levels of proinflammatory cytokines, such as IL-6 and TNF-α, were significantly higher in KO mice than in WT mice (1.76 ± 0.03 pg/ml vs. 1.50 ± 0.04 pg/ml, *P* < 0.01; 1.83 ± 0.04 pg/ml vs. 1.18 ± 0.03 pg/ml, *P* < 0.001, respectively) (Fig. 7). There were no apparent differences in any other proinflammatory cytokines, including IFN-γ, IL-10, IL-12p70, IL-1β, IL-2, IL-4, IL-5, and KC/GRO.

## Discussion

The adipokine irisin is involved in the dysregulation of adipokine secretion. It might lead to the development of excess adiposity and a decrease in insulin sensitivity and the regulation of the inflammatory response^16–19^. In this study, we observed endocrine metabolism in irisin lacking mice. Our results demonstrated that irisin lacking mice showed poor ‘browning response’, glucose/lipid metabolic derangements, and decreased bone strength and bone mass.

WAT is the primary site of triglyceride storage, and BAT specializes in energy expenditure. Irisin is known to mediate the browning of WAT into BAT. BAT has drawn attention as a novel preventive and therapeutic target to treat obesity and metabolic diseases such as T2D. Our study showed an intraperitoneal fat accumulation, and a decreased ‘browning response’ in irisin lacking mice. Furthermore, our results revealed that the KO mice had glucose/lipid metabolic derangements, reduced glucose tolerance, and insulin resistance. These results were consistent with in vivo studies showing that irisin overexpression directly promotes Akt phosphorylation to improve glucose/lipid metabolism and insulin resistance in HFD mice^20^. In addition to the direct effects of irisin, there seem to be two potential reasons for insulin resistance in KO mice. One is that irisin lacking was associated with increased growth hormone (GH) concentration in KO mice (Supplementary Figure 2). GH is known to catabolize stored fat to release energy to promote growth in other tissues. However, recent studies indicated that the chronic stimulation of lipolysis by GH results in an increased free fatty acid (FFA) concentration in the systemic circulation. The excess FFA activates the TGF-β signaling pathway to induce insulin resistance through Smad-3-mediated downregulation of the *Fndc5* gene^21,22^. Hence, a sustained release of high levels of GH significantly contributes to the development of insulin resistance by antagonizing the antilipolytic action of insulin. GH also mediates the deregulation of the FSP27-PPARγ axis, alters adipose tissue homeostasis and contributes to the development of insulin resistance^23^. Another reason is likely that, in our study, irisin deletion was related to the increased level of proinflammatory cytokine (IL-6 and TNF-α) in serum. In adipose tissue, IL-6 and TNF-α were the activators of the NF-κB pathway, linked to insulin resistance in obesity^24,25^. Studies have suggested that IL-6 production in abdominal adipose tissue is threefold higher than that of subcutaneous adipose tissue^25,26^. In the present study, the KO mice had abdominal fat accumulation, which indicated that IL-6 might be one of the factors that make abdominal adipose tissue a high-risk factor for the development of insulin resistance.

In bone metabolism, the results showed that KO mice had decreased bone strength and bone mass at 24 weeks of age, increased bone resorption. Our previous studies revealed that irisin promotes osteoblast proliferation and differentiation via the MAPK signaling pathway and inhibits differentiation in osteoclast precursor cells^11,12^. We also previously found that exogenously administered irisin has potential effects in resisting bone loss in OVX mice^13^. In the present study, irisin lacking was linked to increased osteoclast numbers and increased expression of RANKL, which indicated that irisin deficiency promoted bone resorption and resulted in sharp bone loss in KO mice. However, Kim et al. reported that *Fndc5* null mice had higher trabecular bone mass than wild-type mice^27^. This discrepancy might be due to the animal model. They performed germline deletion of Fndc5(irisin residues 30–69 deletion, 70–112 remaining). In this study, we clipped the 18th, and 19th nucleotides in exon three led to a frameshift mutation, resulting in irisin residues 43–112 deletion. A recent study demonstrated that the flexible region of 55–58 and 106–108 residues, and C-terminal of irisin are vital for its activity^28^. Disrupting the dimerization of irisin might result in a partly reduced effect on cell differentiation. Therefore, our deletion of irisin (43–112 residues) disturbed salt bridges and C-terminal, severely affecting its physiological functions. Additionally, it is known that bone homeostasis of bone is tightly regulated by the balanced activities between bone-resorbing activity of osteoclast cells and bone-forming ability osteoblast cells^29^. This complicated process is governed by many endocrine factors. Rahman et al. presented the beneficial action of BAT and insulin-like growth factor 1(IGF-1) include positive effects on bone. Insulin-sensitive is also positively correlated with bone mass^30^. Thus, in this study, the poor ‘brown response’, insulin resistance, and decreased serum level of IGF-1 (Supplementary Figure 2) were likely related to the reduced bone mass in mice lacking irisin. IGF-1 plays a critical role in developing the growing skeleton by establishing both longitudinal and transverse bone accrual^31^. A previous study indicated that older men with low serum IGF-1 have an increased fracture risk, and the association between serum IGF-1 and fracture risk is partly mediated by BMD^32^. In addition, irisin was an anti-inflammatory factor and reduced the secretion of proinflammatory cytokines (IL-6 and TNF-α) in 3T3 L1 cells^33–36^. In this study, the results showed that irisin lacking was related to the increased levels of IL-6 and TNF-α in KO mice. Firstly, excess IL-6 and TNF-α production increased the osteoclast-covered bone surface and decreased bone formation. Secondly, in the present study, the increased level of IL-6 and TNF-α were linked to a higher expression of RANKL in KO mice, which is consistent with the research showing that inflammation induces bone loss by increasing osteocyte protein expression of RANKL^37,38^.

To our knowledge, this was the first study to observe multiple metabolic processes in irisin lacking mice. We found poor ‘browning response’, hyperlipidemia, insulin resistance, reduced bone mass, and increased inflammatory factors in irisin lacking mice. These results indicated metabolic disorders in irisin lacking mice, suggesting that irisin might be involved in multiple metabolic processes. However, our current results are initial observations, and are unable to elucidate the specific mechanism (e.g., signaling pathway) of irisin. Moreover, metabolism is a complex process; whether there are other factors that play a role together with irisin needs further studies.

## Conclusion

Irisin lacking was related to poor ‘brown response’, glucose/lipid derangement, and decreased bone mass in mice. The increased bone resorption induced bone loss in irisin lacking mice. This study opens the prospect of targeting irisin in improving metabolic derangement and preventing bone loss.

### Supplementary information

Supplementary Information.
