# Author Correction: Disordered metabolism in mice lacking irisin

**DOI:** 10.1038/s41598-021-01412-1

**Published:** 2021-11-04

**Authors:** Yunyao Luo, Xiaoyong Qiao, Yaxian Ma, Hongxia Deng, Charles C. Xu, Liangzhi Xu

**Affiliations:** 1 Reproductive Endocrinology and Regulation Laboratory West China Second University Hospital, Sichuan University, #20 Section 3, Ren Min Nan Road, Chengdu, 610041 Sichuan People’s Republic of China; 2 The Joint Laboratory for Reproductive Medicine of Sichuan University, The Chinese University of Hong Kong, Hong Kong, People’s Republic of China; 3 Key Laboratory of Birth Defects and Related Diseases of Women and Children (Sichuan University), Ministry of Education, Chengdu, People’s Republic of China; 4 Department of Obstetrics and Gynecology, West China Second University Hospital, Sichuan University, Chengdu, People’s Republic of China; 5 College of Engineering, The Ohio State University, Columbus, OH USA

Correction to: *Scientific Reports* 10.1038/s41598-020-74588-7, published online 15 October 2020

The original version of this Article contained errors in Figure 6 where the arrows were incorrectly placed, and in Figure 7 where the data of TNF-α in WT and KO mice was incorrect.

The original Figures 6 and 7 and accompanying legends appear below.

The original Article has been corrected.

**Figure 6:**
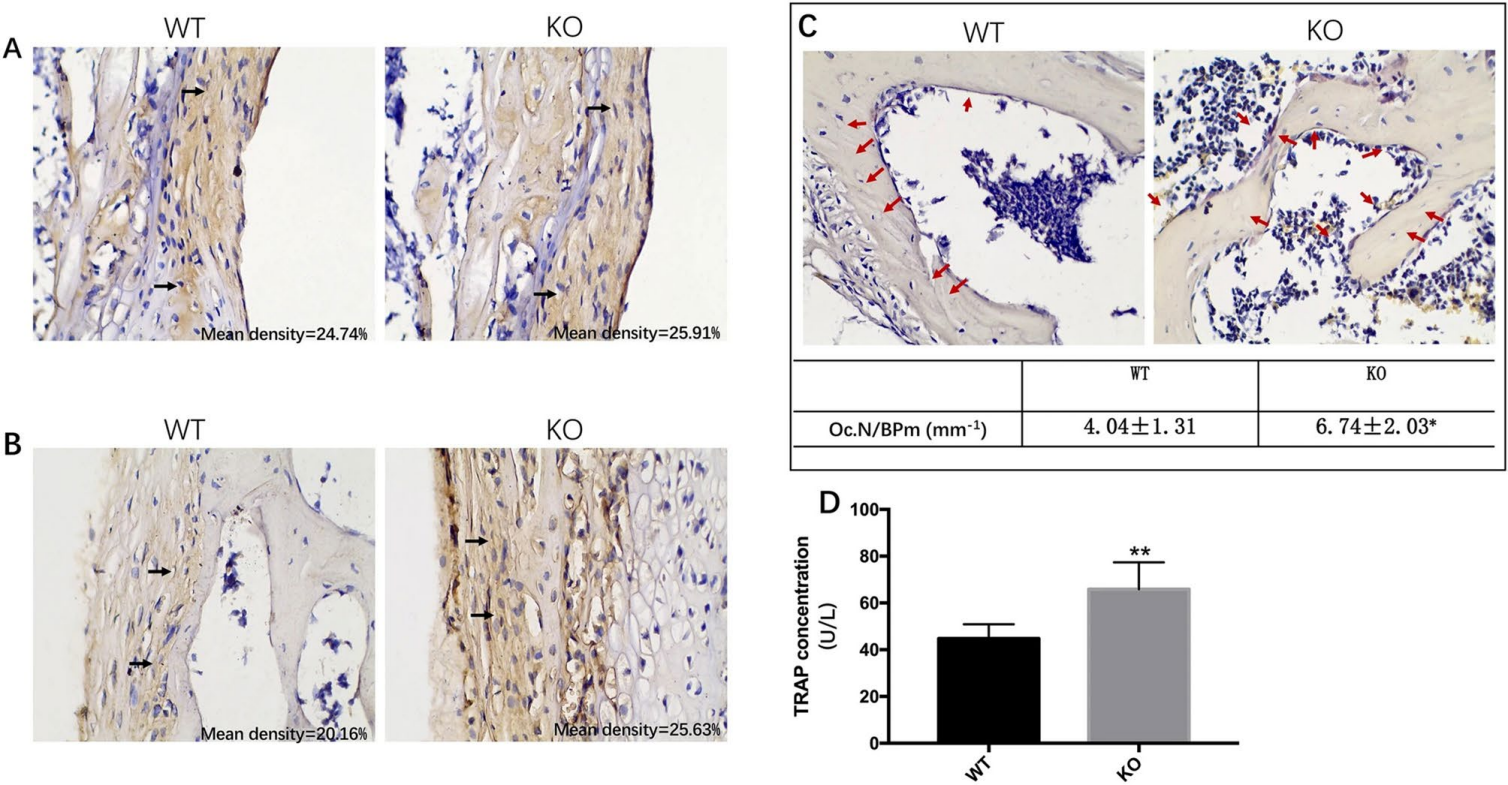
Increased bone resorption in irisin lacking mice. (**A**, **B**) Sections of the distal shaft of the femur were stained with osteoprotegerin (OPG) (1:60) and receptor activator of nuclear factor-kB ligand (RANKL) (1:80) antibodies. Positive (black arrows) osteoblasts are shown, 200 × . (**C**) Representative images of the distal metaphyseal region of the femur, together with cell counts (osteoclasts) per bone perimeter (Bpm). Red arrows, tartrate-resistant acid phosphatase (TRAP)-positive osteoclasts, 100 ×, (NIS-Elements Viewer, v4.2.0; https://www.downza.cn/soft/2751-21.html); (**D**) serum levels of osteocalcin TRAP (with three replicates). (Graphpad Prism, v7.0, https://www.xue51.com/soft/3932.html). Data are presented as the mean ± SEM (n = 15 per group). *P < 0.05, **P < 0.01 compared to the WT group.

**Figure 7:**
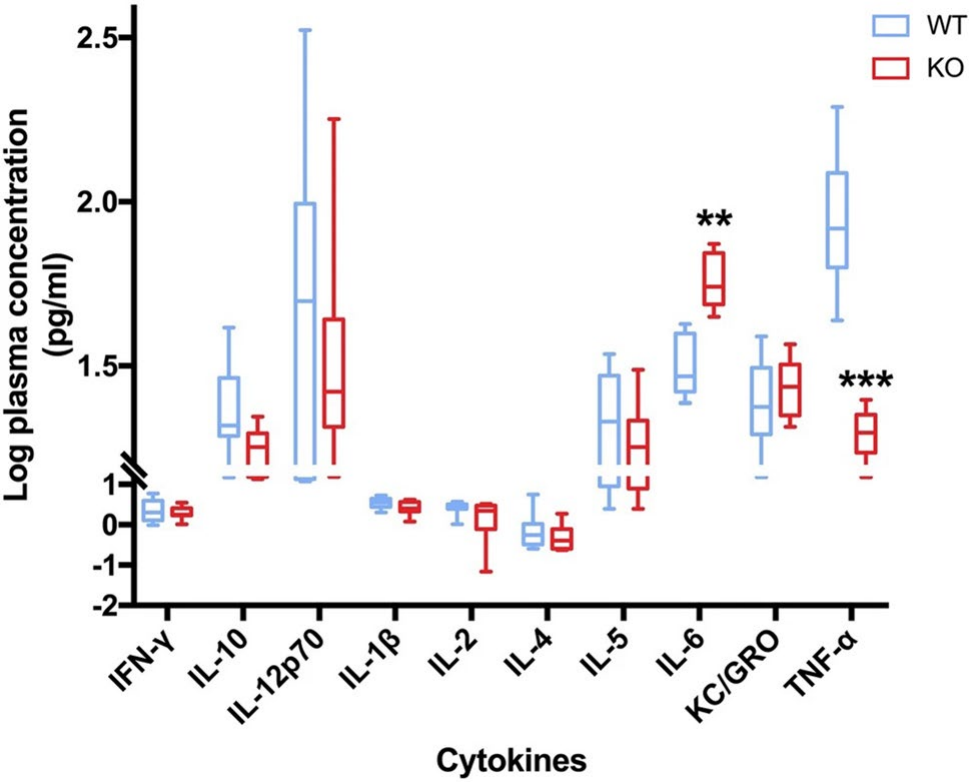
The distribution of the log of transformed cytokine concentrations. The figure represents the distributions of interferon-gamma (IFN-γ), interleukin 10 (IL-10), interleukin 12p70 (IL-12), interleukin 1-beta (IL-1β), interleukin 2 (IL-2), interleukin 4 (IL-4), interleukin 5 (IL-5), interleukin 6 (IL-6), growth-regulating oncogenes (GRO), and tumor necrosis factor-alpha (TNF-α). (n = 15 per group, with three replicates). (Graphpad Prism, v7.0, https://www.xue51.com/soft/3932.html). **P < 0.01, *P* < 0.001 compared to the WT group.

