# Author Correction: Disordered metabolism in mice lacking irisin

**DOI:** 10.1038/s41598-023-45919-1

**Published:** 2023-11-09

**Authors:** Yunyao Luo, Xiaoyong Qiao, Yaxian Ma, Hongxia Deng, Charles C. Xu, Liangzhi Xu

**Affiliations:** 1 Reproductive Endocrinology and Regulation Laboratory West China Second University Hospital, Sichuan University, #20 Section 3, Ren Min Nan Road, Chengdu, 610041 Sichuan People’s Republic of China; 2 The Joint Laboratory for Reproductive Medicine of Sichuan University, The Chinese University of Hong Kong, Hong Kong, People’s Republic of China; 3 Key Laboratory of Birth Defects and Related Diseases of Women and Children (Sichuan University), Ministry of Education, Chengdu, People’s Republic of China; 4 Department of Obstetrics and Gynecology, West China Second University Hospital, Sichuan University, Chengdu, People’s Republic of China; 5 College of Engineering, The Ohio State University, Columbus, OH USA

Correction to: *Scientific Reports* 10.1038/s41598-020-74588-7, published online 15 October 2020

The original version of this Article contained errors in Table 1, where the value of ‘iBAT ratio (%)’ was erroneously stated as ‘Liver ratio (%)’ and ‘Spleen ratio (%)’ in the column ‘WT’.

The correct and incorrect values appear below.

Incorrect:

**Table:** 

	WT
Liver ratio (%)	0.011 ± 0.0002
Spleen ratio (%)	0.007 ± 0.0002

Correct:

**Table:** 

	WT
Liver ratio (%)	0.054 ± 0.01
Spleen ratio (%)	0.070 ± 0.0002

The original Article has been corrected.

