# Supplementary material for: Disordered metabolism in mice lacking irisin
Source: Sci Rep. 2020 Oct 15;10:17368. doi: 10.1038/s41598-020-74588-7 (PMC7567109; doi:10.1038/s41598-020-74588-7)
Supplement: Supplementary file 1 — Supplementary Information. [file 41598_2020_74588_MOESM1_ESM.docx]

**Disordered metabolism in mice lacking irisin**

Yunyao Luo^1,2,3,4, *^, Xiaoyong Qiao^1,2,3,4, *^, Yaxian Ma^1,2,3,4^, Hongxia Deng^1,2,3,4^, Charles C. Xu^5^, Liangzhi Xu^1,2,3,4, *^

^1^Reproductive Endocrinology and Regulation Laboratory West China Second University Hospital, Sichuan University

^2^The Joint Laboratory for Reproductive Medicine of Sichuan University–The Chinese University of Hong Kong, People’s Republic of China.

^3^Key Laboratory of Birth Defects and Related Diseases of Women and Children (Sichuan University), Ministry of Education, P.R. China.

^4^Department of Obstetrics and Gynecology, West China Second University Hospital, Sichuan University, Chengdu, People’s Republic of China.

^5^College of Engineering, The Ohio State University, Columbus, Ohio, USA.

*Yunyao Luo and Xiaoyong Qiao contributed equally to this work.

^*^Address correspondence to: Liangzhi Xu, PhD, West China Second University Hospital of Sichuan University, #20 Section 3, Ren Min Nan Road, Chengdu, Sichuan, China, 610041. E-mail: xuliangzhi_art@126.com, Tel: 86-28-85501346.

Grant supporters: Scientific Research Projects of The National Natural Science Fund (81671421).


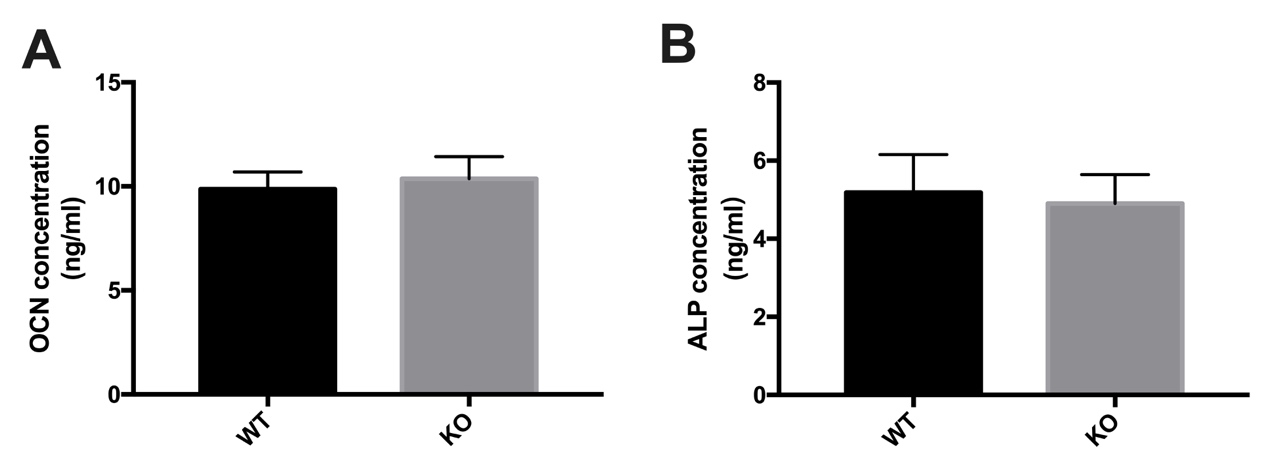


**FIG. S1.** Osteocalcin (OCN) and [Alkaline Phosphatase](https://www.elabscience.cn/p-mouse_balp(bone_alkaline_phosphatase)_elisa_kit-48454.html)(ALP) levels in irisin lacking mice. (A) OCN and (B) ALP. (Graphpad Prism, v7.0, <http://www.xue51.com/soft/3932.html>). Data presented the mean±SD (n=15 per group).


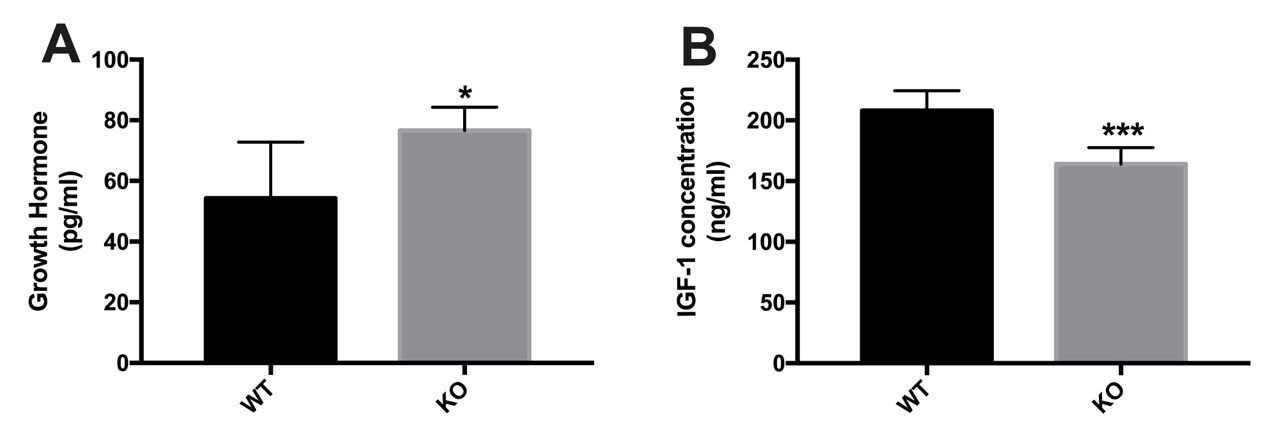


**FIG. S2.** Growth hormone (GH) and insulin-like growth factor I (IGF-1) concentrations in irisin lacking mice. (A) GH and (B) IGF-1. (Graphpad Prism, v7.0, <http://www.xue51.com/soft/3932.html>). Data presented the mean±SD (n=15 per group); *P<0.05, ***P<0.001 compared to WT mice.

**The explanation for Figure 1C**


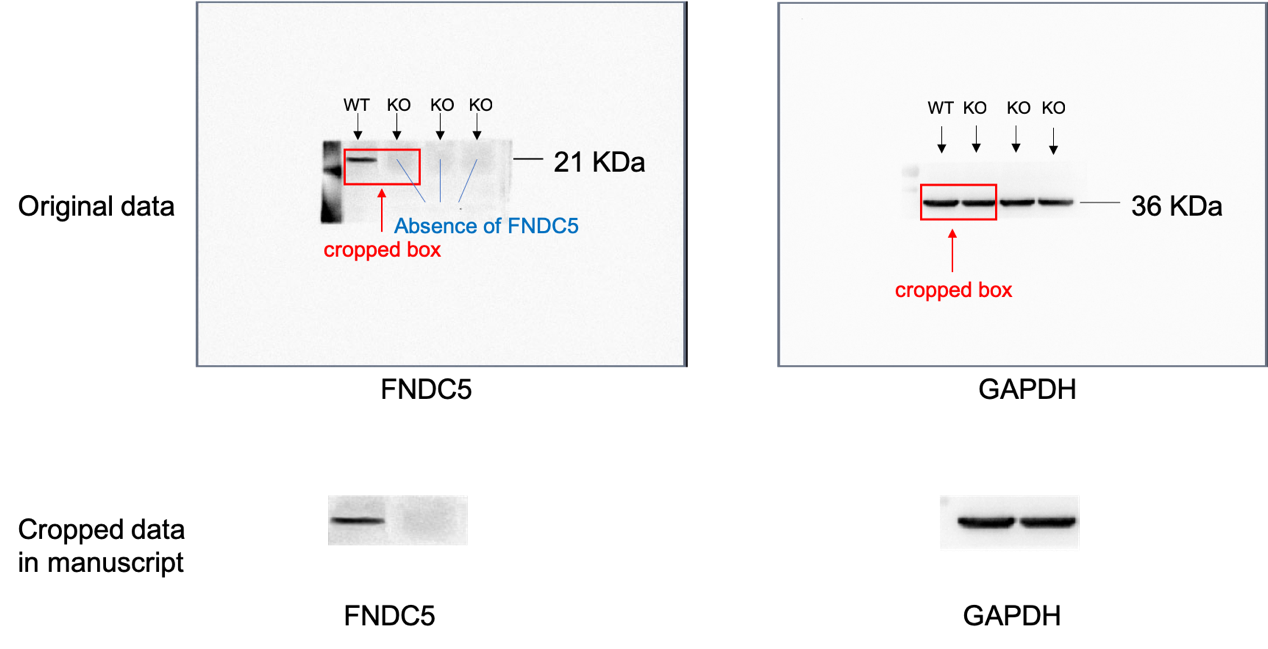


**The original data for Figure 1C**


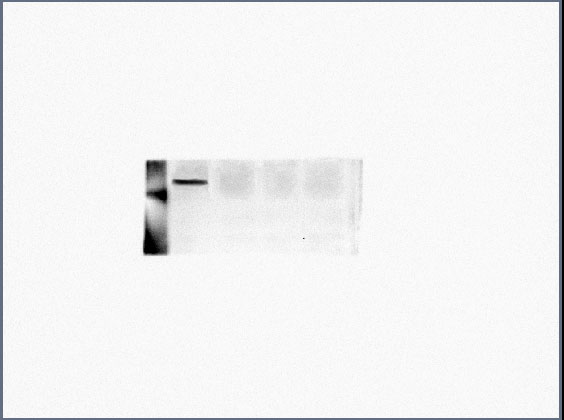

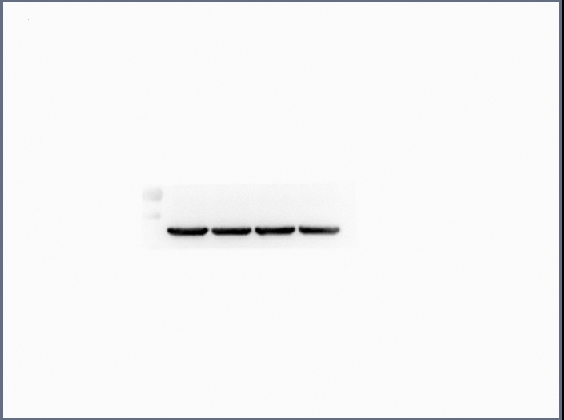


FNDC5 GAPDH
